# Safety and efficacy of molnupiravir in SARS‐CoV‐2‐infected patients: A real‐life experience

**DOI:** 10.1002/jmv.28011

**Published:** 2022-08-02

**Authors:** Andrea De Vito, Agnese Colpani, Alessandra Bitti, Beatrice Zauli, Maria Chiara Meloni, Marco Fois, Lucia Denti, Sara Bacciu, Claudia Marcia, Ivana Maida, Sergio Babudieri, Giordano Madeddu

**Affiliations:** ^1^ Unit of Infectious Diseases, Department of Medicine, Surgery and Pharmacy University of Sassari Sassari Italy

**Keywords:** antiviral treatment, COVID‐19, COVID‐19 treatment, early treatment, molnupiravir, SARS‐CoV‐2

## Abstract

Since the start of the severe acute respiratory syndrome coronavirus‐2 (SARS‐CoV‐2) pandemic, several treatments have been proposed to cure coronavirus disease 2019 (COVID‐19) and prevent it. Molnupiravir is a ribonucleoside prodrug of *N*‐hydroxycytidine with an in vitro and in vivo activity against SARS‐CoV‐2. We conducted a retrospective cohort study that included all people treated with molnupiravir between January 10, 2022, and March 31, 2022, at the University Hospital of Sassari. Molnupiravir was prescribed, according to the Italian Agency of Drug indications, to patients with recent symptom onset (≤5 days), no need for oxygen supplementation, and with a high risk of disease progression for the presence of chronic diseases. We included 192 people with a mean age of 70.4 ± 15.4 years, with 144 (75%) patients over 60 years. During the follow‐up, 20 (10.4%) patients showed a disease progression. At the multivariate analysis, older age, having neurological disease, having dyspnea at the onset of the symptoms, and acquiring SARS‐CoV‐2 infection during hospital admission were associated with an increased risk of progression. In contrast, an early start of treatment was associated with a reduced risk of disease progression. Molnupiravir was also extremely safe since 13 (6.8%) adverse events were reported, with only one interruption. Our study shows that monlupiravir confirmed its efficacy and safety in a real‐life cohort that included a high percentage of elderly people with a high comorbidity burden.

## 1 INTRODUCTION

Since the start of the severe acute respiratory syndrome coronavirus‐2 (SARS‐CoV‐2) pandemic in December 2019, infected people have reached half billion, with more than six million deaths.^1^

SARS‐CoV‐2, especially the B.1.1.529 (omicron) variant, has a short incubation period (2–11 days); the virus enters the host cells using the angiotensin‐converting enzyme 2 receptors and starts its replication.^2^, ^3^, ^4^ Then, symptoms appear, and in particular, the most common symptoms include fever, cough, and dyspnea, while minor symptoms include gastrointestinal disorders, anosmia, dysgeusia, headache, and skin lesions.^5^, ^6^, ^7^ After symptom onset, the disease can progress to life‐threatening systemic inflammation, respiratory failure, and multiorgan dysfunction.

Since the start of the pandemic, several treatments have been proposed for COVID‐19 (e.g., steroids, heparin, antivirals, and monoclonal antibodies)^8^, ^9^ to prevent the infection (vaccine, monoclonal antibodies)^10^ and the progression of the disease (antiviral, monoclonal antibodies).^11^, ^12^

Molnupiravir (EIDD‐2801) is a ribonucleoside prodrug of *N*‐hydroxycytidine (NHC) that showed activity against SARS‐COV‐2 both in vitro and in vivo, with a high genetic barrier,^13^ and excellent safety. After the Phase 2 trials, the selected dose was 800 mg every 12 h for 5 days.^14^

However, no real‐life data are available. Therefore, we decided to conduct a retrospective cohort study to evaluate the efficacy and safety of this new drug in a real‐life setting.

## 2 METHODS

All patients who have been evaluated by our team, in the University Hospital of Sassari, between January 10, 2022, and March 31, 2022, were retrospectively included in the analysis. Inclusion criteria were: (i) age ≥18 years; (ii) confirmed diagnosis of SARS‐CoV‐2 infection by polymerase chain reaction on nasal–pharyngeal swab; (iii) 5‐day course treatment with molnupiravir.

People with SARS‐CoV‐2 infection in the emergency room were evaluated by one of our eight young residents in infectious disease (A. C., A. B., B. Z., M. C. M., M. F., L. D., S. B., and C. M.), who collected anamnesis, medical history, and performed a clinical evaluation. Then, if people were eligible for molnupiravir, according to international guidelines and the Italian Agency of Drug indications, a second evaluation was performed by an expert doctor (A. D. V., I. M., S. B., and G. M.).

Molnupiravir was prescribed, according to the Italian Agency of Drug indications, to patients with recent symptom onset (≤5 days), no need for oxygen supplementation, and with a high risk of disease progression for the presence of at least one of the following chronic diseases: (i) obesity (body mass index > 30); (ii) diabetes mellitus with organ damage or hemoglobin A1c > 7.5%; (iii) kidney failure; (iv) severe lung disease; (v) severe cardiovascular disease; (vi) immune deficiency; (vii) cancer. Contraindication included (i) estimated glomerular filtration rate < 30 ml/min/1.73 m^2^; (ii) pregnancy; (iii) advanced chronic liver disease. In addition, male patients should accept to use condoms for at least 3 months if their partner was fertile, while fertile female patients should accept to use condoms for at least 4 days since the end of treatment.

Also, we collected medical history, symptoms, computer tomography (CT) findings (if performed), blood test results (if performed), the necessity of hospital admission, the reason for it, data regarding disease progression (O_2_ supplementation, noninvasive ventilation, invasive ventilation, death), date of hospital discharge, and date of first negative SARS‐CoV‐2 test. In addition, the Charlson Comorbidity Index (CCI) score and 4C score were calculated when all information was available.

We defined early treatment as less than 4 days (0–3 days) between the onset of symptoms and the start of treatment.

The study's endpoint was to evaluate the safety and efficacy of the antiviral treatment to avoid the disease progression (defined as the necessity to start oxygen supplementation, noninvasive ventilation, and death due to COVID‐19). In addition of the disease and to evaluate possible predictors of disease progression.

### 2.1 Statistical analysis

Quantitative variables were summarized with medians and 25th−75th percentiles (interquartile range), whereas qualitative ones by absolute and relative (percentages) frequencies. Shapiro–Wilk test was used to assess the normality of quantitative data. Subgroup differences in quantitative variables were evaluated by the Mann–Whitney test. Pearson's *χ* ^2^ or Fisher's exact tests were used to assess differences for qualitative variables. Cases were matched with controls by age and sex (1:1). Logistic regression analysis was performed to identify factors associated with 28‐day mortality. Two‐tailed *p* value less than 0.05 was considered statistically significant. All statistical analyses were carried out with STATA version 16.1 (StatsCorp).

### 2.2 Ethical issues

This research was included in the protocol “COVID‐19‐SS,” approved by the Local Ethical Committee of the University Hospital of Cagliari (PG/2020/9411).

## 3 RESULTS

Since January 10, 2022, our team evaluated more than 1000 people with SARS‐CoV‐2, but only 192 met the criteria to start molnupiravir treatment. The mean age of people treated with molnupiravir was 70.4 ± 15.4 years, with 144 (75%) patients over 60 years. Regarding comorbidities, the most common were cardiovascular disease (96, 50.0%), chronic lung disease (56, 29.2%), obesity (51, 26.6%), and active cancer (51, 26.6%). The majority of patients had at least one dose of SARS‐CoV‐2 vaccination, but only 109 (56.8%) had a complete cycle of vaccination (three doses, or a second dose in the last 120 days before infection). Forty‐three (22.7%) patients acquired the infection in a hospital ward where they were admitted for other medical conditions. Among them, most patients (39, 93%) received antiviral treatment in the first 3 days since the symptoms' onset versus 78% of people evaluated in the emergency room and 85% as outpatients.

Most of the patients were treated in January, during which Italy had the maximum SARS‐CoV‐2 incidence since the start of the pandemic.

**Figure 1:**
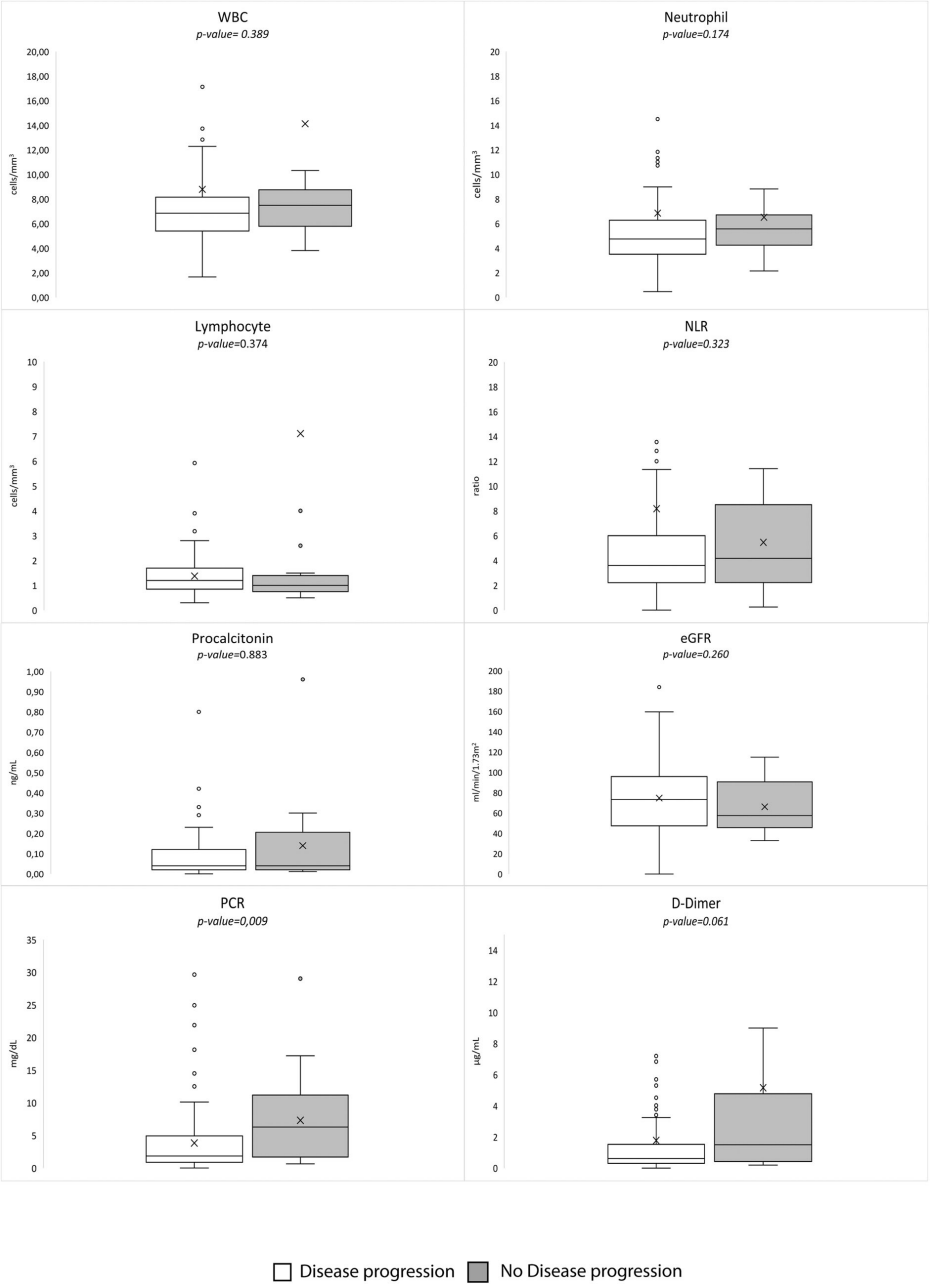
Blood test results in 90 SARS‐CoV‐2‐infected people treated with molnupiravir, divided between people with (16 patients) and without (64 patients) disease progression. CRP, C‐reactive protein; eGFR, estimated glomerular filtration rate; NLR, neutrophil–lymphocyte ratio; SARS‐CoV‐2, severe acute respiratory syndrome coronavirus‐2; WBC, white blood cells.

During the follow‐up, 20 (10.4%) patients underwent disease progression. These patients had a higher CCI score and 4C score; they had dyspnea at symptom onset and had a higher prevalence of pulmonary consolidation at the CT scan (Table 1). Regarding blood tests, only C‐reactive protein was significantly higher among patients with disease progression (Figure 1).

**Table 1:** Characteristics of 192 people with SARS‐CoV‐2 infection treated with molnupiravir, divided between people with (20 patients) and without (172 patients) disease progression

	Overall (192 patients)	Progression (20 patients)	No progression (172 patients)	*p* Value*
Age (years), mean ± SD	70.4 ± 15.4	79.9 ± 10.1	69.3 ± 15.6	0.0033
Male gender, *n* (%)	78 (59.5)	11 (55)	97 (56.4)	0.905
Comorbidities, *n* (%)				
BMI > 30	51 (26.6)	6 (30)	45 (26.2)	0.713
CKD	19 (9.9)	2(10)	17 (9.9)	1
Immune deficiency	36 (18.7)	1 (5)	35 (20.4)	0.131
Diabetes	41 (21.4)	4 (20)	37 (21.5)	1
Liver diseases	12 (6.2)	2 (10)	10 (5.8)	0.362
Chronic lung diseases	56 (29.2)	10 (50)	46 (26.7)	0.030
Hemoglobinopathy	4 (2.1)	1 (5)	3 (1.7)	0.358
Neurological disorders	26 (13.5)	7 (35)	19 (11.1)	0.003
Cancer	51 (26.6)	7 (35)	44 (25.6)	0.367
Cardiovascular disorders	96 (50.0)	11 (55)	85 (49.4)	0.637
CCI, mean ± SD	4.73 ± 2.18	6.1 ± 2.05	4.58 ± 2.15	0.0029
4C score, mean± SD (data available for 120 patients)	7.73 ± 3.38	10.47 ± 2.87	7.28 ± 3.25	0.0002
Early molnupiravir treatment, *n* (%)^a^	160 (83.3)	12 (60)	148 (86.1)	0.003
Vaccine (at least one dose), *n* (%)	171 (89.1)	16 (80)	99 (90.1)	0.244
Last vaccine dose 14–120 before symptom onset, *n* (%)	109 (56.8)	7 (35)	102 (59.3)	0.038
Symptoms, *n* (%)				
Fever	87 (45.3)	9 (45)	78 (45.4)	0.976
Cough	109 (56.8)	11 (55)	98 (57.0)	0.866
Dyspnea	32 (16.7)	11 (55)	21 (12.2)	<0.0001
Sore throat	66 (34.4)	4 (20)	62 (34.4)	0.214
Asthenia	85 (44.3)	7 (35)	78 (45.4)	0.378
Headache	47 (24.5)	3 (15)	44 (25.6)	0.414
GS symptoms	25 (13.0)	3 (15)	22 (12.8)	0.729
CT findings, *n* (%) (data available for 107 patients)				
GGO	28/107 (26.2)	5/17 (29.4)	23/90 (25.6)	0.740
Pulmonary consolidation	17/107 (15.9%)	7/17 (41.2)	10/90 (11.1)	0.002
Months of infection, *n* (%)				0.011
January	80 (41.7)	14 (70)	66 (38.4)	
February	54 (28.1)	5 (25)	49 (28.5)	
March	58 (30.2)	1 (5)	57 (33.1)	
Hospital infection	43 (22.7)	9 (45)	34 (20.1)	0.012
Other prophylactic treatment, *n* (%)				
Casirivimab/imdevimab 600 mg/600 mg	54 (28.1)	13 (65)	41 (23.8)	<0.001
Sotrovimab 500 mg	31 (16.1)	1 (5)	30 (17.44)	0.208
Death, *n* (%)	13 (7.2)	6 (30)	7 (4.4)	<0.0001

Abbreviations: BMI, body mass index; CCI, Charlson Comorbidity Index; CKD, chronic kidney disease; CT, computer tomography; GGO, ground glass opacities; SARS‐CoV‐2, severe acute respiratory syndrome coronavirus‐2; SG, gastrointestinal; SD, standard deviation.

^a^ Treatment was started 0–3 days after symptom onset

* *p* Value was calculated with Student's *t* test, *χ* ^2^ test, or Fisher's exact test as appropriate.

Overall, 13 (7.2%) patients died, but only six of them died because of COVID‐19. In the other seven patients, the infection occurred while staying in the hospital for other medical conditions. In particular, three had metastatic cancer, two had a cerebrovascular accident, one had sepsis from a urinary tract infection, and one had severe heart failure. No COVID‐19 progression was reported in any of these patients.

Regarding the factor associated with disease progression, in the multivariate analysis, older age, having a neurological disease, and having dyspnea at symptom onset was associated with an increased risk of progression. On the contrary, an early start of treatment was associated with a reduced risk of disease progression. In the univariate analysis, pulmonary consolidation at the CT scan, a higher CCI score, a higher 4C score, and being treated with casirivimab/imdevimab were associated with an increased risk of progression (Table 2), while acquiring the infection in March and having received the last vaccination dose between 14 and 120 days before symptom onset was associated with lower risk of disease progression.

**Table 2:** Logistic regression analysis to assess the relationship between clinical characteristics and progression of disease in 192 patients with SARS‐CoV‐2 infection treated with molnupiravir

	Univariate analysis			Multivariate analysis		
	OR	95% CI	*p* Value	aOR	95% CI	*p* Value
Age	1.06	1.02–1.10	0.005	1.06	1.01–1.12	0.018
Chronic lung diseases	2.74	1.07–7.01	0.036	1.63	0.46–5.76	0.466
Neurological disorders	4.34	1.54–12.2	0.002	5.12	1.17–22.40	0.030
CCI score	1.39	1.11–1.74	0.004			
4C score^a^	1.42	1.16–1.75	0.001			
Last vaccine dose 14–120 before symptoms onset	0.37	0.14–0.97	0.044	1.39	0.38–5.12	0.614
Early molnupiravir treatment^b^	0.24	0.09–0.66	0.005	0.10	0.02–0.47	0.004
Dyspnea	8.79	3.26–23.7	<0.0001	8.04	2.15–90.01	0.002
Pulmonary consolidation^c^	5.6	1.74‐18.01	0.004			
CRP^a^	1.08	1.01–1.16	0.033			
d‐Dimer^a^	1.08	1.00–1.17	0.040			
Hospital infection	3.32	1.27–8.65	0.014	2.76	0.69–10.95	0.149
Month of infection						
January	(ref.)					
February	0.48	0.16–1.42	0.187			
March	0.08	0.11–0.65	0.018	0.10	0.01–1.25	0.074
Casirivimab/imdevimab	5.93	2.22–15.87	<0.001	3.21	0.81–12.72	0.098
Sotrovimab	0.25	0.03–1.93	0.184			

Abbreviations: aOR, adjusted odd ratio; CCI, Charlson Comorbidity Index; CI, confidence interval; CRP, C‐reactive protein; OR, odds ratio; ref., reference; SARS‐CoV‐2, severe acute respiratory syndrome coronavirus‐2.

^a^ Data are available for 122 patients.

^b^ Treatment was started 0–3 days after symptom onset.

^c^ Data are available for 107 patients.

Thirteen (6.8%) patients reported adverse events. In particular, three patients had diarrhea, three patients complained of dizziness, one patient had diarrhea, nausea, and dizziness, two people had a rash, two patients reported metallic taste, and one patient had abdominal pain with increased transaminases level; only in the latter patient, the treatment was interrupted.

## 4 DISCUSSION

Molnupiravir is a ribonucleoside prodrug of NHC, which targets SARS‐CoV‐2 RNA‐dependent RNA polymerase. After oral administration, NHC is phosphorylated intracellularly to NHC triphosphate, which is incorporated by RNA SARS‐CoV‐2 polymerase into the viral RNA. After the incorporation, it misdirects the viral polymerase to incorporate either adenosine or guanosine during the viral replication, causing an accumulation of deleterious errors in the viral genome that render the virus noninfectious and unable to replicate.^15^, ^16^, ^17^ In addition, molnupiraivr showed a high genetic barrier in different in vitro studies, and it remains active against the different SARS‐CoV‐2 variants, while different monoclonal antibodies (e.g., casirivimab/imdevimab, bamlanivimab) have a reduced activity against SARS‐CoV‐2 B.1.1.529 variant.^18^

The advantage of molnupiravir is that it could easily be administered at home, while monoclonal antibodies and remdesivir need to be administered in a hospital setting, leading to many organizational issues and higher costs. Furthermore, monlupiravir has no interaction with other chronic treatments; thus, it could be more easily prescribed compared with nirmatrelvir/ritonavir.^11^

Molnupiravir has been investigated in the Phase 3 trial MOVe‐OUT;^19^ a total of 1433 people were enrolled, of whom 716 were treated with molnupiravir and 717 with placebo. In the group of people treated with molnupiravir, only 7.3% were hospitalized or had died versus 14.1% in the placebo group, with a relative risk reduction of 31%.^19^

In our analysis, the percentage of people with a disease progression was higher than in the MOVe‐OUT trial (10.4% vs. 7.3%). However, the older age of our patients could explain it since the median age in people treated with molnupiravir in the MOVE‐OUT trial was 42 years, while in our study, it was 70.4 years. Also, the comorbidities were different; indeed, in the trial, obesity was the principal comorbidity (75.1%), followed by diabetes (14.9%) and serious heart condition (12%). Furthermore, in our study, having neurodegenerative disorders was associated with an increased risk of disease progression, but no data about this condition was reported in the MOVE‐OUT trial. Another important difference between our study and MOVE‐OUT trial is that in our cohort, 43 (22.7%) patients acquired the infection in the hospital, where they have been admitted for other medical conditions, and nine of them (20.9%) had a clinical progression.

Comparing our results with EPIC‐HR and PINETREE trials that analyzed, respectively, the efficacy of nirmatrelvir/ritonavir and early remdesivir, our study showed a higher percentage of disease progression.^20^, ^21^ In particular, less than 1% of the patients were hospitalized or died in the EPIC‐HR trial, but no data about the presence of comorbidities were reported. The median age in the EPIC‐HR trial was 45 years in respect of 70.4 years in our study. In the PINETREE trial, the percentage of hospitalized patients was less than 1%, but the mean age was 50 years old. The wide age difference between our study and the two trials may explain the difference in the percentage of patients with disease progression. Age has been consistently shown to be the major predictor of disease progression and mortality in COVID‐19 patients.^22^, ^23^

Early treatment with molnupiravir was demonstrated to reduce significantly the risk of disease progression (odds ratio: 0.10; 95% confidence interval: 0.02–0.47; *p* = 0.004) in contrast with the MOVE‐OUT trial, where only people who started treatment after 4–5 days since the onset of the symptoms had better outcomes when compared with placebo. In our population, the effect of early treatment was evident also in complicated patients such as people who get the infection during hospitalization. Among them, four people did not receive early treatment, and two of them had a disease progression; on the contrary, among the 39 people who received early treatment, only 7 had a disease progression (50% vs. 17.9%).

Our data support an early treatment start, especially in patients with a higher comorbidity burden and older age, underrepresented in the MOVE‐OUT trial.

The majority of people who had a disease progression have been infected in the months of January (*p* = 0.011); the possible explanation is that in Italy in January there was still a significant percentage of the SARS‐CoV‐2 variant B.1.617.2 (delta), which has a higher fatality compared to the variant B.1.1.529 (Omicron).^24^

In our study, people without SARS‐CoV‐2 vaccination or a complete vaccination cycle (more than 120 days since the last dose) showed the same risk of disease progression. Our data support the efficacy of molnupiravir also in older people without SARS‐CoV‐2 vaccination.

Regarding safety, in our study, the percentage of adverse events was lower in respect of the MOVE‐OUT trial (6.8% vs. 8.0%), while the percentage of discontinuation was similar (0.76% vs. 0.6%),^19^ thus confirming the high tolerability and safety of monlupiravir in clinical practice.

Our study has some limitations that should be addressed. First, this is an observational, retrospective study. Second, our experience is monocentric, and this could not entirely reflect the international situation. Finally, CT scans and blood tests were performed only in a part of the patients treated with molnupiravir.

## 5 CONCLUSION

Our study shows the efficacy and safety of molnupiravir in a real‐life cohort that included a high percentage of elderly people with a high comorbidity burden. However, more studies are needed to evaluate the impact of molnupiravir in the progression of COVID‐19, identify the best timing to start it, and compare it to other early treatments.

## AUTHOR CONTRIBUTIONS

*Conceptualization*: Andra De Vito and Giordano Madeddu. *Investigation*: Agnese Colpani, Alessandra Bitti, Beatrice Zauli, Maria Chiara Meloni, Marco Fois, Lucia Denti, Sara Bacciu, and Claudia Marcia. *Formal analysis*: Andrea De Vito and Giordano Madeddu. *Methodology*: Andrea De Vito, Sergio Babudieri, and Giordano Madeddu. *Validation*: Ivana Maida, Sergio Babudieri and Giordano Madeddu. *Visualization*: Alessandra Bitti, Beatrice Zauli, Marco Fois, Ivana Maida, and Claudia Marcia. *Resources*: Maria Chiara Meloni, Lucia Denti, and Sara Bacciu. *Writing—original draft*: Andrea De Vito, Agnese Colpani, and Giordano Madeddu. *Writing—review and editing*: Alessandra Bitti, Beatrice Zauli, Maria Chiara Meloni, Marco Fois, Lucia Denti, Sara Bacciu, Claudia Marcia, Ivana Maida, and Sergio Babudieri.

## CONFLICT OF INTEREST

The authors declare no conflict of interest.

## Data Availability

The data that support the findings of this study are available from the corresponding author upon reasonable request.
